# Altered brain network centrality in middle‐aged patients with retinitis pigmentosa: A resting‐state functional magnetic resonance imaging study

**DOI:** 10.1002/brb3.1983

**Published:** 2020-12-09

**Authors:** Qi Lin, Fei‐Ying Zhu, Yong‐Qiang Shu, Pei‐Wen Zhu, Lei Ye, Wen‐Qing Shi, You‐Lan Min, Biao Li, Qing Yuan, Yi Shao

**Affiliations:** ^1^ Department of Ophthalmology Jiangxi Province Ocular Disease Clinical Research Center The First Affiliated Hospital of Nanchang University Nanchang China; ^2^ Department of Radiology The First Affiliated Hospital of Nanchang University Nanchang China

**Keywords:** functional magnetic resonance imaging, network centrality, resting state, retinitis pigmentosa

## Abstract

**Objective:**

The purpose of this study is to explore the underlying functional network brain activity changes of patients in middle‐aged with retinitis pigmentosa (RP) and the relationships with clinical features such as depression scale and visual functioning using voxel‐wise degree centrality (DC) method.

**Methods:**

We included 16 patients with RP (11 men, 5 women) and 16 healthy controls (HCs; 11 men, 5 women). Participants were matched in terms of age, weight, gender and handedness (age and weight between the two groups were compared using independent sample *t*‐tests, gender and handedness were compared using chi‐square test). We use the voxel‐wise DC method to assess spontaneous brain activity. Receiver operating characteristic (ROC) curve analysis was performed to distinguish between RP patients and HCs. Correlation analysis was used to examine the relationships between mean DC values in various brain regions and clinical features (such as depression scale and visual functioning) in RP patients.

**Results:**

Compared with HCs, the DC values of patients with RP were reduced in the right medial frontal gyrus, bilateral cuneus, bilateral precuneus, and bilateral superior frontal gyrus, and increased in the right cerebellum posterior lobe, left inferior temporal gyrus, and right fusiform gyrus. The mean DC values in the bilateral cuneus negatively correlated with the depression scale, and those in the bilateral precuneus positively correlated with the Visual Functioning Questionnaire‐25.

**Conclusions:**

Middle‐aged patients with RP exhibit abnormal brain network activity in various brain regions, and this may underlie the pathological mechanism of RP.

## Graphical abstract

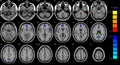

## 1 INTRODUCTION

Retinitis pigmentosa (RP) is a heterogeneous group of inherited retinal degeneration diseases characterized by constricted visual fields and photoreceptor cell dysfunction and apoptosis (Yang, Peng, et al., 2018). The main clinical manifestations of RP are chronic progressive visual field loss, night blindness, pigmented retinopathy, electroretinogram abnormalities, and color vision abnormalities, eventually leading to decreased vision. Also known as hereditary retinal dystrophy, it affects 1 in every 4,000 people in the United States and approximately 1 in 5,000 worldwide, making RP the most common inherited disease of the retina (O'Neal & Luther, 2018). It is usually bilateral, but there have been reports of unilateral eye involvement. While RP may present and progress with a variety of clinical manifestations, the first symptom is generally nyctalopia, or loss of night vision, which is followed by gradual visual field narrowing (O'Neal & Luther, 2018). Depending on disease severity and the rate of progression, tunnel vision or complete vision loss can be the result (O'Neal & Luther, 2018). Postpolar cataract is a common late‐stage complication of RP. The crystal turbidity is star‐shaped, and it lies in the inferior cortex of the posterior capsule. Although progression is slow, and eventually, the whole crystal becomes turbid.

Functional magnetic resonance imaging (fMRI) has been widely used to appraise neurophysiological damage in many eye diseases (Shi et al., 2020). Previous studies have confirmed that hemispherical synchronization is closely related to visual experience (Foubert et al., 2010; Mima et al., 2001) .In general, studies have shown that disturbances in the eye and visual pathway can significantly impact the structure of gray and white matter in the brain (O'Neal & Luther, 2018).

The brain is composed of complex large‐scale networks characterized by inter‐regional interactions (Yan et al., 2010) .Voxel‐wise degree centrality (DC) is applied to measure functional connectivity (FC) at the voxel level in human brain connectivity groups (Di Martino et al., 2013). Unlike amplitude of low‐frequency fluctuation (ALFF; Di Martino et al., 2013; Huang, Cai, et al., 2015; Tan et al., 2015, 2016; Zuo et al., 2012) and regional homogeneity (ReHo) techniques (Huang, Zhong, et al., 2015), the DC method does not need to define regions of interest (ROIs). This makes it a better network metric because it calculates the number of direct connections for a given voxel in the network and reflects its functional connections in the brain network without a priori selection. DC has been used to examine the neuropathological mechanisms of many diseases including autism (De Pasquale et al., 2013) and Parkinson’s disease (Shao et al., 2015). Here, we examined changes in functional network brain activity in RP patients and investigated their relationship with clinical features such as depression scale and visual functioning.

## 2 MATERIALS AND METHODS

### 2.1 Subjects

Sixteen patients with RP, (11 men, 5 women) were recruited from the Ophthalmology Department of the First Affiliated Hospital of Nanchang University Hospital in Jiangxi province of China. The inclusion criteria for RP were as follows: (a) diagnosed with RP based on relevant medical history, symptoms, and visual function check and ophthalmoscopy; (b) no abnormality of the cerebral parenchyma on cranial MRI; and (c) no other ocular diseases in either eye (glaucoma, cataracts, amblyopia, optic neuritis, strabismus, etc.). The exclusion criteria were: (a) eye diseases, trauma or ophthalmic surgery; (b) mental illness (depression, paranoia), cardiovascular disease, brain disease (cerebral hemorrhage, cerebral infarction, cerebral vascular malformation); or (c) abnormality of cerebral parenchyma on cranial MRI.

Sixteen healthy controls (HCs; 11 males, 5 females) were recruited from Nanchang in Jiangxi Province in China. They were matched with the RP group in terms of gender, age, weight, and handedness. All HCs met the following criteria: (a) no eye disease; (b) no mental illness (depression, paranoia); (c) ability to undergo MRI scans (e.g., no metal devices in the body).

All research protocols were written in accordance with the Helsinki Declaration. All subjects participated voluntarily and understood the purpose, content, and risks of the study before providing written consent.

### 2.2 MRI data acquisition

A Siemens Trio 3.0 T scanner (produced in Munich, Germany) with an 8‐channel phased‐array head coil was applied to perform MRI scanning of all subjects as previously described (Cai et al., 2015). The functional data were acquired by a 3D spoiled gradient recalled‐echo pulse sequence with the following parameters: 176 structural images (acquisition matrix = 256×256, field of view = 250×250 mm, echo time = 2.26 ms, repetition time = 1,900 ms, thickness = 1.0 mm, gap = 0.5 mm, flip angle = 9°). We also obtained 240 functional images (acquisition matrix = 64×64, field of view = 220×220 mm, thickness = 4.0 mm, gap = 1.2 mm, repetition time = 2,000 ms, echo time = 30 ms, flip angle = 90°, 29 axial).

### 2.3 Functional magnetic resonance imaging data analysis

MRIcro software (University of Nottingham) was used to classify functional data and remove incomplete data. The remaining data were preprocessed by DPARSFA software (Institute of Psychology, CAS), including digital imaging and communication form conversion in medicine, slice timing, head motion correction, spatial normalization, and Gaussian kernel of full width at half maximum (FWHM) of 6 × 6 × 6 mm^3^ at half maximum. Subjects with a maximum displacement >1.5 mm in *x*, *y*, or *z* and 1.5° angular motion were not included in the analysis. Friston's six head motion parameters can be used to eliminate head motion effects, which makes it more reasonable to speculate that higher‐order models may be more effective in eliminating head motion effects. We also performed linear regression to remove other artifacts, including signals from ROIs of the ventricle and regions centered on white matter. Using the standard echo‐planar imaging template, the fMRI images were spatially normalized to the Montreal Neurological Institute space after head motion correction. The fast Fourier transform was applied to convert the time series of the blood‐oxygen level dependent signal to the frequency domain. Then the square root of the power spectrum was calculated and averaged across 0.01–0.08 Hz for every voxel. More data analysis details were described in a previous study (Chao‐Gan & Yu‐Feng, 2010).

### 2.4 Degree centrality

On the basis of the individual voxel function network, we generated DC values by calculating the number of significant super‐threshold correlations (or degrees of binary adjacency matrices) for each subject. Using the following equation (Di Martino et al., 2013), each individual's voxel DC map was converted to a *z*‐score graph: Zi¼DCi – mean (DC of all voxels in brain mask)/*SD* (DC of all voxels in brain mask).

### 2.5 Statistical analysis

We compared the clinical variables and demographic of both groups using independent sample *t*‐tests and chi‐square test in SPSS software version 20.0 (IBM Corp.; Before statistical analysis, the Shapiro–Wilk test was used to verify that the clinical variables and demographic data between the two groups obeyed the normal distribution according to the condition that the W value is close to 1 and the significant level is >.05.). Differences were considered statistically significant at *p* < .05. At the same time, functional data were compared with two‐sample *t*‐tests using REST software. The statistical threshold of voxel level for multiple comprehensive comparisons was set at *p* < .05 after using Gaussian random field theory. Alphasim corrected at a cluster size >40 voxels and *p* < .01.

We generated receiver operating characteristic (ROC) curves to classify the mean DC values regions of the cerebrum distinct between the RP and HC groups. Clinical Parameters and Data collections The association DC and clinical features of RP patients should be evaluated though separate linear regression models which included age, gender and Duration of RP(y) as nuisance covariates. Correlation analysis was performed to assess the relationships between DC and clinical features of RP patients.

### 2.6 Brain‐behavior correlation analysis

REST software was used to classify encephalic regions with DC differences between the two groups as ROIs, after which the mean DC value was calculated for each by averaging all voxels. The relationship between behavioral performance such as depression scale and visual functioning and the mean DC value in each ROI was analyzed using linear correlation analysis in the RP group. Differences were considered statistically significant at *p* < .05.

### 2.7 Clinical data analysis

Cumulative clinical measurements (RP duration and best‐corrected visual acuity [VA] and intraocular pressure [IOP] of each eye) of RP patients were recorded and analyzed with independent sample *t*‐tests (differences significant at *p* < .05).

## 3 RESULTS

### 3.1 Demographics and behavioral results

No statistically significant differences were observed between two groups in age (*p* = .781), weight (*p* = .836) or IOP (*p* = .639 and *p* = .779 for right and left, respectively) as shown in Table 1. The mean ± standard deviation (*SD*) of RP duration was 12.43 ± 5.41 years. The details are presented in Table 1.

**Table 1:** Demographics and clinical measurements by groups

Condition	RP	HC	*t*	*p*‐value
Male/female	11/5	11/5	N/A	>.99
Age (years)	49.21 ± 6.19	50.35 ± 5.93	0.092	.781
Weight (kg)	61.25 ± 6.67	59.17 ± 5.59	0.089	.836
Handedness	16R	16R	N/A	>.99
Duration of RP(ys)	12.43 ± 5.41	N/A	N/A	N/A
Best‐corrected Va‐right eye	0.42 ± 0.13	1.02 ± 0.19	−0.532	.005
Best‐corrected Va‐left eye	0.51 ± 0.15	1.05 ± 0.23	−0.679	.007
IOP‐R (mmHG)	17.72 ± 1.89	16.14 ± 1.53	0.083	.639
IOP‐L (mmHG)	19.21 ± 2.43	19.57 ± 2.09	0.072	.779

Abbreviations: HC, healthy control; L, left; N/A, not applicable; R, right; RP, retinitis pigmentosa.

* *p* < .05 Independent *t*‐tests comparing two groups.

### 3.2 Degree centrality differences

**Figure 1:**
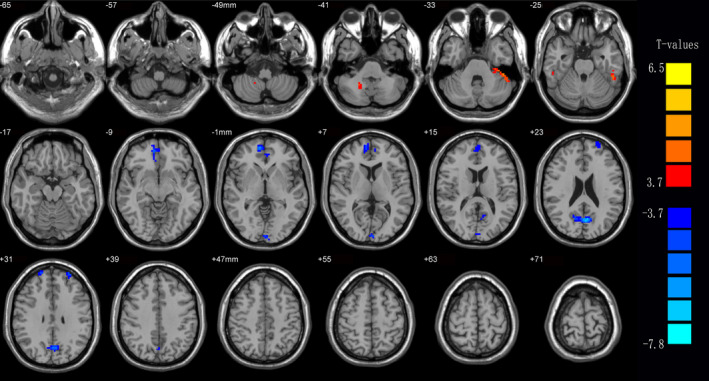
Voxel‐wise comparison of DC in the RP and HC groups. Significant differences in DC were observed the right Medial Frontal Gyrus, bilateral Cuneus, the right Medial Frontal Gyrus, bilateral Precuneus, the left Superior Frontal Gyrus and the right Superior Frontal Gyrus, the right Cerebellum Posterior Lobe, the left Inferior Temporal Gyrus, and the right Fusiform Gyrus. The red areas denote higher DC values, the blue indicates lower DC values. *p* < .05 for multiple comparisons using Gaussian Random Field (GRF) theory (*z* > 2.3, cluster‐wise *p* < .05 corrected). Abbreviations: DC, Degree centrality; RP, retinitis pigmentosa; HC, healthy controls

**Figure 2:**
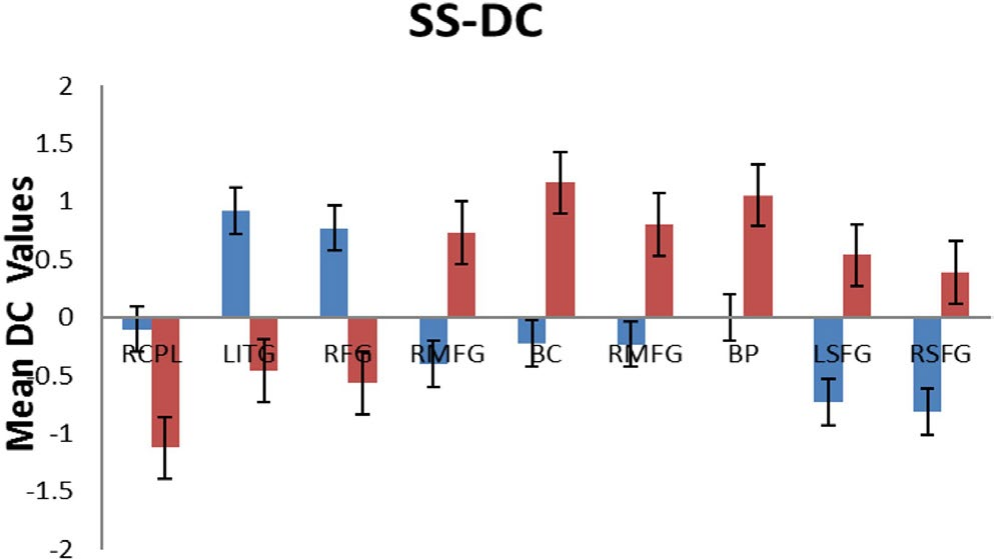
The mean of altered DC values between the RP patients and HCs. Abbreviations: DC, degree centrality; RP, retinitis pigmentosa; HCs, health controls; RCPL, right cerebellum posterior lobe; LITG, left Inferior Temporal Gyrus; RFG, right Fusiform Gyrus; RMFG, right Medial Frontal Gyrus; BC, bilateral Cuneus; BP, bilateral Precuneus; LSFG, left Superior Frontal Gyrus; RSFG, right Superior Frontal Gyrus

Compared to HCs, middle‐aged patients with RP showed significantly reduced DC values in the right medial frontal gyrus, bilateral cuneus, bilateral precuneus, and bilateral superior frontal gyrus (Figure 1 [blue] and Table 2) and significantly increased DC values in the right cerebellum posterior lobe, left inferior temporal gyrus, and right fusiform gyrus (Figure 1 [red] and Table 2). The mean values of DC changes between groups are displayed in Figure 2. No obvious correlation was observed between mean DC values and behavioral performance in any brain region in the RP group (*p* > .05).

**Table 2:** Brain regions with significant differences in DC between RP patients and HCs

Brain areas	MNI coordinates			BA	Peak voxels	*T* value	ROI
	*X*	*Y*	*Z*				
SS < HC							
R Medial frontal gyrus	12	54	−3	10	102	−5.9565	4
B Cuneus	0	−96	3	18	35	−5.0242	5
R Medial frontal gyrus	9	51	18	10	41	−4.5595	6
B Precuneus	−6	−69	27	31	136	−7.4784	7
L Superior frontal gyrus	−27	57	30	10	28	−4.7941	8
R Superior frontal gyrus	21	60	33	10	21	−4.8026	9
SS > HC							
R Cerebellum posterior lobe	21	−60	−39		22	4.4325	1
L Inferior temporal gyrus	−51	−39	−30		90	6.5114	2
R fusiform gyrus	39	−30	−27		21	4.7322	3

The statistical threshold was set at voxel level with *p* < .05 for multiple comparisons using Gaussian Random Field (GRF) theory (*z* > 2.3, cluster‐wise *p* < .05 corrected).

Abbreviations: BA, Brodmann area; DC, Degree centrality; HCs, healthy controls; L, left; MNI, Montreal Neurological Institute; R, right; RP, retinitis pigmentosa.

### 3.3 Correlation analysis

**Figure 3:**
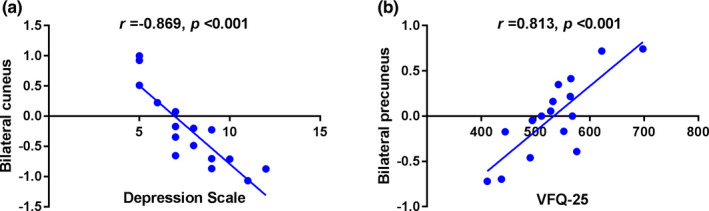
Correlations between the mean DC values of different regions and the clinical behaviors in RP group. In the RP group, （a）the mean DC value in the bilateral Cuneus was negatively correlated with the depression scale (r = −.869, p < .001), and ( b )the mean DC value in the bilateral Precuneus were positively correlated with the Visual Functioning Questionnaire‐25 (r = .813, p < .001). Abbreviations: DC, degree centrality; RP, retinitis pigmentosa

In the RP group, the mean DC value in the bilateral cuneus was negatively correlated with the depression scale (*r* = −.869, *p* < .001), and the mean DC value in the bilateral precuneus were positively correlated with the Visual Functioning Questionnaire‐25 score (*r* = .813, *p* < .001; Figure 3).

### 3.4 Receiver operating characteristic curves

**Figure 4:**
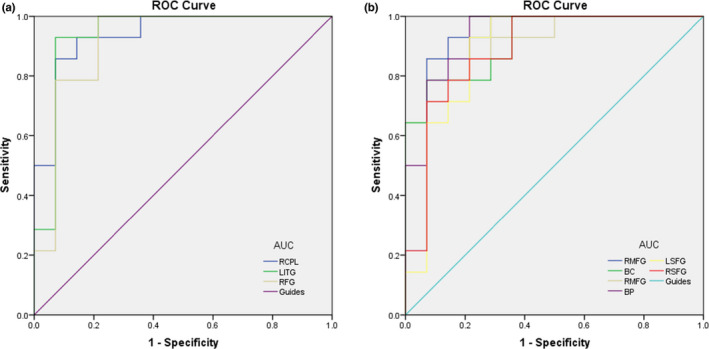
Receiver operating characteristic curve. Notes: (a) The area under the ROC curve were 0.939, (*p* < .001; 95% CI: 0.852–1.000) for RCPL, LITG 0.939 (*p* < .001; 95% CI: 0.837–1.000), RFG 0.913 (*p* < .001; 95% CI: 0.795–1.000). (b) The area under the ROC curve were 0.923 (*p* < .001; 95% CI: 0.810–1.000) for RMFG, BC 0.918 (*p* < .001; 95% CI: 0.820–1.000), RMFG 0.913 (*p* < .001; 95% CI: 0.812–1.000), BP 0.939 (*p* < .001; 95% CI: 0.853–1.000), LSFG 0.888 (*p* < .001; 95% CI: 0.754–1.000), RSFG 0.888 (*p* < .001; 95% CI: 0.760–1.000). Abbreviations: ROC, receiver operating characteristic. RCPL, right cerebellum posterior lobe; LITG, left Inferior Temporal Gyrus; RFG, right Fusiform Gyrus; RMFG, right Medial Frontal Gyrus; BC, bilateral Cuneus; BP, bilateral Precuneus; LSFG, left Superior Frontal Gyrus; RSFG, right Superior Frontal Gyrus

Regions with DC values that were significantly different between groups were identified with ROC curves. The area under the ROC curve (AUC) indicated the diagnostic cutoff. In this study, the AUCs were clearly shown in Table 3 and Figure 4.

**Table 3:** The area under the ROC curve (AUC)

Brain areas	AUC	*p*	95% CI
Right cerebellum posterior lobe	0.939	<.001	0.852–1.000
Left inferior temporal gyrus	0.939	<.001	0.837–1.000
Right fusiform gyrus	0.913	<.001	0.795–1.000
Right medial frontal gyrus	0.923	<.001	0.810–1.000
Bilateral cuneus	0.918	<.001	0.820–1.000
Right medial frontal gyrus	0.913	<.001	0.812–1.000
Bilateral precuneus	0.939	<.001	0.853–1.000
Left superior frontal gyrus	0.888	<.001	0.754–1.000
Right superior frontal gyrus	0.888	<.001	0.760–1.000

Abbreviations: AUC, the area under the ROC curve; ROC, receiver operating characteristic.

## 4 DISCUSSION

**Figure 5:**
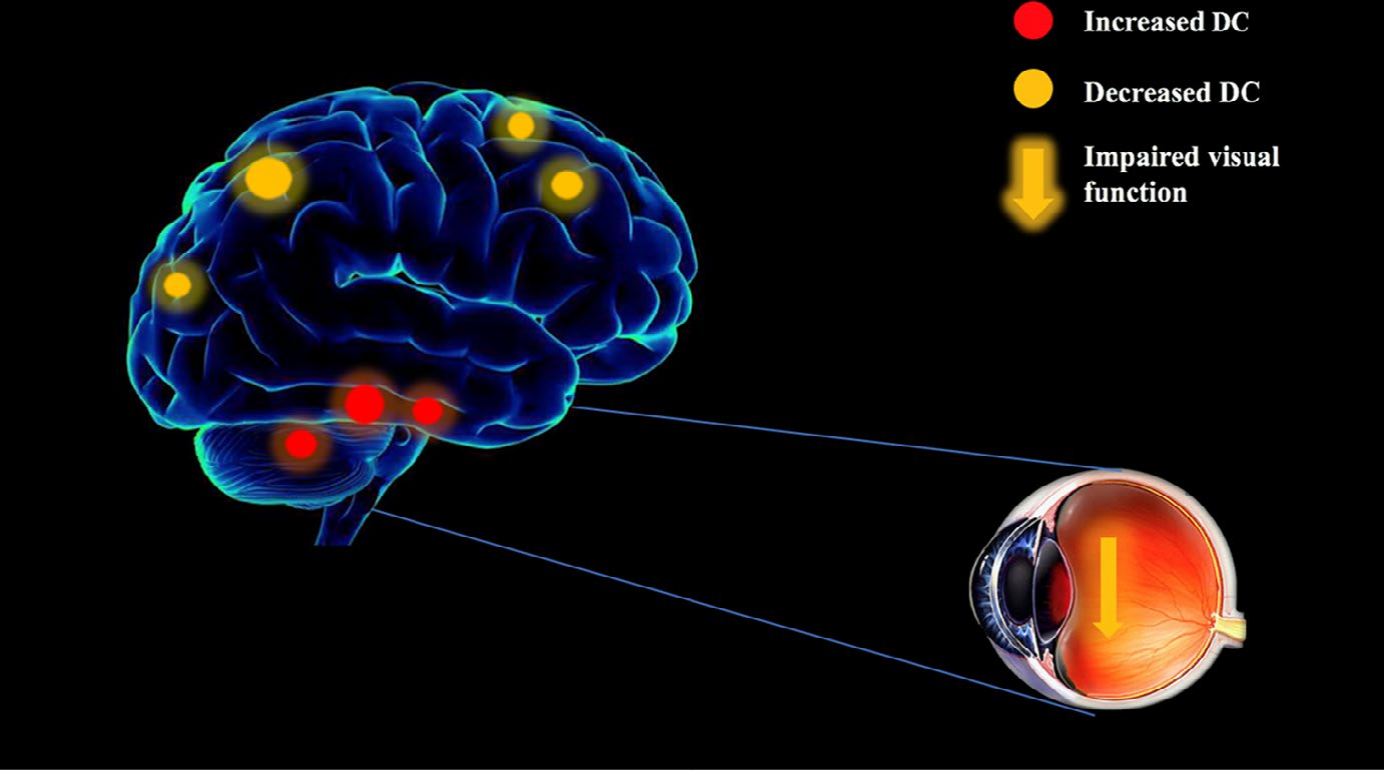
The DC results of brain activity in the RP group. Compared with the HCs, the DC of the following regions were decreased to various extents: 1‐ R Medial Frontal Gyrus (*t* = −5.9565), 2‐ B Cuneus (*t* = −5.0242) and 3‐ R Medial Frontal Gyrus (*t* = −4.5595), 5‐ B Precuneus (*t* = −7.4784), 6‐ L Superior Frontal Gyrus (*t* = −4.7941), 7‐ R Superior Frontal Gyrus (*t* = −4.8026) and decreased DC values in the 8‐ R Cerebellum Posterior Lobe (*t* = 4.4325), 9‐ L Inferior Temporal Gyrus (*t* = 6.5114), 10‐ R Fusiform Gyrus (*t* = 4.7322). Notes: The sizes of the spots denote the degree of quantitative changes. Abbreviations: DC, degree centrality; RP, retinitis pigmentosa; R. right; L, left; B, bilateral

To our knowledge, this is the first study to investigate functional network brain activity using DC methods in middle‐aged patients with RP. We found that this group showed significantly reduced DC values compared to HCs in the right medial frontal gyrus, bilateral cuneus, bilateral precuneus, and bilateral superior frontal gyrus and significantly increased DC values in the right cerebellum posterior lobe, left inferior temporal gyrus, and right fusiform gyrus (Figure 5).

### 4.1 Analysis of reduced degree centrality values in adult RP

We know that the medial frontal gyrus plays an important role in cognitive and attentional function (Tops & Boksem, 2011).The frontal lobe is involved in optical positioning, eye rotation adjustment, fixation and spatial information processing. The function of the superior and middle gyrus is related to the movement of the body, language and higher thinking activities. The superior frontal gyrus is thought to consist of several cytoarchitecturally different subregions including BA6, 8, 9, and 32 (Petrides & Pandya, 1999, 2002). Significant reactions related to visual cues of color or shape have been reported in BA8 (Komatsu, 1982). Although the origin of this neurological activity is not known, it occurs in both BA8 and BA6. Previous studies have shown that some frontal regions, incuding the middle frontal gyrus, are associated with spontaneous activity in the primary visual area (Wang et al., 2008).In our study, the RP group showed lower DC values in the right midfrontal gyrus and bilateral superior frontal gyrus, suggesting that the above‐mentioned brain regions of RP patients are damaged, and the functional connection with primary visual area is reduced, causing visual impairment and visual impairment Adverse consequences.

The cuneus located in the medial occipital gyri is part of the occipital lobe, corresponding to Brodmann area (BA) 17. The cuneus receives significant input from the lower visual field (Cohen, 2011) corresponding to the contralateral superior retina and is responsible for processing visual information. (Atapour et al., 2017; Vinje & Gallant, 2000) The white matter tracts pass through the cuneus to connect the precuneus with the parietal lobe (Parker et al., 2014). The precuneus has been proven to be a part of the default mode network (Cunningham et al., 2016). The default mode network is a region of the brain network that involves self‐referencing thoughts. In the restless state of the human brain, certain functional activities continue. Studies have found that connectivity from the precuneuscan be altered in major depression through the cuneus (Yang, Zhang, et al., 2018). Studies have also shown that cuneus may be neuroimaging markers of depression (Yao et al., 2018).We found that patients with RP have significantly lower DC values in this region compared to HC, which may be related to chronic progressive visual field loss and decreased vision in RP patients. In addition, our study showed that in the RP group, the mean DC value of bilateral cuneus was negatively correlated with the depression scale, which further confirmed the relationship between wedge leaves and depression, which may be a neuroimaging marker of depression.

The precuneus (BA7) is a part of the superior parietal lobule (Wallentin et al., 2008) that plays a critical role in visuospatial cognitive tasks (Cavanna & Trimble, 2006; Knauff et al., 2003; Suchan et al., 2002). The precuneus has been suggested to participate in visual space imaging (Cavanna & Trimble, 2006), self‐processing (Nagahama et al., 1999), situational memory retrieval, (Lundstrom et al., 2005) spatial location encoding (Frings et al., 2006), and the default mode network (Utevsky et al., 2014). In addition, damage to the precuneus and posterior cingulate can result in a unique condition (Hecaen & Ajuriaguerra, 1954) called Balint’s syndrome that has a cardinal feature, “which is the inability to perceive the visual field as a whole, despite intact visual fields, during simple confrontation with single small stimuli” (Raichle et al., 2001). We observed decreased DC in the right precuneus in the RP group, which may be associated with vision loss.

### 4.2 Analysis of increased degree centrality values in adult RP

Previous studies on the function of the cerebellum have focused on regulating balance and muscle tone. Lizette et al. (2015) have found that the visual cortex and cerebellar function in blind patients are reduced. The cerebellar posterior lobe dominates cognition and emotion. The V1 lobules of the cerebellum are related to spatial visual tasks and have extensive fiber crossings with the brain. Our study showed that the DC value of the posterior cerebellar lobe was increased in patients with RP, and the functional connectivity of the posterior cerebellar lobe in the RP group was enhanced compared with HCs. We speculate that this is a compensatory performance of the posterior cerebellar lobe to maintain equivalent behavior. The performance further confirmed the role of the posterior cerebellum in vision. It should also be noted that the cerebellum is considered to be closely related to the limbic system and also plays an important role in emotional cognitive processing. An early study suggested that posterior leafy lesions could severely affect spatial memory, emotional regulation, and executive function (Wu, 2014). It is well known that RP can affect vision. Visual impairment can cause a range of social and emotional problems (Evans et al., 2007). Long‐term visual impairment can severely affect mental health and reduce quality of life. One‐third of people with visual impairment have clinical symptoms of depression (Fenwick et al., 2012). Therefore, we should also pay attention to the negative emotions of RP patients.

The inferior temporal gyrus is below the middle temporal gyrus, and is connected posteriorly with the inferior occipital gyrus. In humans, it is also known as the IT cortex (Kolb & Whishaw, 2014) since it is located in a specific region of the human temporal lobe (Gross, 2008). The IT cortex processes the visual stimuli of objects in our field of vision, which involves perceiving and processing visual stimuli amplified in the V1, V2, V3 and V4 regions of the occipital lobe (Kolb & Whishaw, 2014). This region also is responsible for processing object color and form. The current results revealed significantly higher DC values in the inferior temporal gyrus in the RP group. These findings suggest that inferior temporal gyrus dysfunction might be related to progressive visual field loss, night blindness, and electroretinogram and color vision abnormalities in RP.

The fusiform gyrus is part of the temporal and occipital lobes in BA37, also known as the occipitotemporal gyrus, which is involved in processing color information. Ramachandran (Ramachandran, 2011) reported that the angular gyrus is involved in color processing, and the fusiform gyrus transmits information during this process. The fusiform gyrus is also connected to the visual pathway because there cross‐activation between the angular gyrus and fusiform curl (Hubbard & Ramachandran, 2005). In the current study, DC values in the right fusiform gyrus were significantly higher in the RP group. This may underlie color vision dysfunction in patients with RP.

## 5 CONCLUSION

Our results demonstrate abnormal spontaneous activity in many brain regions of middle‐aged patients with RP. These events may be related to the pathological mechanism of RP. Measuring brain activity changes might be a useful clinical indicator to monitor RP. The correlation analyses suggested that changes in these areas could be related to the depression and the visual function in the RP group.

## 6 PROSPECTS AND LIMITATIONS

The DC method is a useful technique for monitoring whole‐brain activity. It has also been successfully applied in some ophthalmological diseases, as mentioned in Table 4. Future research should be performed to clarify the brain regions involved in RP and possibly develop new treatments. However, the current study had several limitations that should be considered. First, the sample size was small; future research should expand the size for more accurate results. Second, the clinical characteristics were not strict, patients with RP were at different stages of disease, which may have affected the accuracy of the results. Future research should distinguish between different stages of RP to more accurately assess brain function activity changes. It would also be important to streamline the scanning protocol. For some subject, the scan time was too long, and slight head movement during certain scans may also can affect DC findings. Despite these shortcomings, the present study of retinitis pigmentosa (RP) of middle‐aged revealed that dysfunction in specific brain areas may underlies the pathogenesis of RP.

**Table 4:** DC method applied in ophthalmological diseases

Author (year)	Disease	Increased DC	Decreased DC
Wang et al. (2017)	Acute unilateral open globe injury	Bilateral primary visual cortex (V1/V2) and left PCUN	Right insula, left insula, RIPL/SMG, IPL/SMG, right supplementary motor area and right postcentral gyrus.
Tan et al. (2018)	Adult comitant exotropia strabismus	Right superior temporal gyrus, bilateral anterior cingulate, right superior temporal gyrus, and left inferior parietal lobule	Right cerebellum posterior lobe, right inferior frontal gyrus, right middle frontal gyrus and right superior parietal lobule/primary somatosensory cortex (S1)
Hu et al. (2018)	High myopia	Right cerebellum posterior lobe, left precentral gyrus/postcentral gyrus, and right middle cingulate gyrus	Right inferior frontal gyrus/insula, right middle frontal gyrus, and right supramarginal/inferior parietal lobule
Zhu et al. (2020)	Trigeminal neuralgia	Right lingual gyrus, right postcentral gyrus, left paracentral lobule, and bilateral inferior cerebellum.	/
Wang et al. (2019)	Diabetic nephropathy and retinopathy	BP	RITG, LSG
Liu et al. (2019)	Exophthalmos of Primary Hyperthyroidism	/	Cerebellum posterior lobe
Hu et al. (2018)	Ophthalmectomy	Left cerebellum posterior lobe, left middle frontal gyrus1, right supramarginal gyrus, left middle frontal gyrus2, right middle frontal gyrus	Left lingual gyrus, bilateral lingual lobe, left cingulate gyrus

Abbreviations: BP, bilateral precuneus; DC, Degree centrality; LSG, left subcallosal gyrus regions; PCUN, precuneus; RIPL, right inferior parietal lobule; RITG, right inferior temporal gyrus; SMG, supramarginal gyrus.

## CONFLICT OF INTEREST

This was not an industry supported study. The authors report no conflicts of interest in this work.

## AUTHOR CONTRIBUTIONS

Qi Lin designed the study and supervised the data collection. Qi Lin and Yi Shao initiated the study and drafted the manuscript. Fei‐Ying Zhu, Yong‐Qiang Shu, Pei‐Wen Zhu, Lei Ye, Wen‐Qing Shi, You‐Lan Min, Biao Li, Qing Yuan, Yi Shao was involved in data collection. Yi Shao critically revised the manuscript. All authors read and approved the manuscript.

## Funding information

This work was supported by the National Natural Science Foundation of China (No:81660158, 81460092, 81400372); Natural Science Key Project of Jiangxi Province (No: 20161ACB21017); Youth Science Foundation of Jiangxi Province (No: 20151BAB215016, 20161BAB215198); Key Research Foundation of Jiangxi Province (No: 20151BBG70223, 20181BBG70004); Education Department Key Project of Jiangxi Province (No: GJJ160020); Teaching Reform of Degree and Graduate Education Research Project of Jiangxi Province (No:JXYJG‐2018‐013); Grassroots Health Appropriate Technology “Spark Promotion Plan” Project of Jiangxi Province (No:20088003); Health Development Planning Commission Science Foundation of Jiangxi Province (No: 20175116); Health Development Planning Commission Science TCM Foundation of Jiangxi Province (No:20150823).

### Peer Review

The peer review history for this article is available at https://publons.com/publon/10.1002/brb3.1983.

## Data Availability

The data that support the findings of this study are available from the corresponding author upon reasonable request.
